# Language related differences of the sustained response evoked by natural speech sounds

**DOI:** 10.1371/journal.pone.0180441

**Published:** 2017-07-20

**Authors:** Christina Siu-Dschu Fan, Xingyu Zhu, Hans Günter Dosch, Christiane von Stutterheim, André Rupp

**Affiliations:** 1 Institut für Theoretische Physik, Heidelberg, Germany; 2 Storz Medical AG, Tägerwilen, Switzerland; 3 Department for General and Applied Linguistics, University of Heidelberg, Heidelberg, Germany; 4 Section of Biomagnetism, Department of Neurology, University of Heidelberg, Heidelberg, Germany; Kyoto University, JAPAN

## Abstract

In tonal languages, such as Mandarin Chinese, the pitch contour of vowels discriminates lexical meaning, which is not the case in non-tonal languages such as German. Recent data provide evidence that pitch processing is influenced by language experience. However, there are still many open questions concerning the representation of such phonological and language-related differences at the level of the auditory cortex (AC). Using magnetoencephalography (MEG), we recorded transient and sustained auditory evoked fields (AEF) in native Chinese and German speakers to investigate language related phonological and semantic aspects in the processing of acoustic stimuli. AEF were elicited by spoken meaningful and meaningless syllables, by vowels, and by a French horn tone. Speech sounds were recorded from a native speaker and showed frequency-modulations according to the pitch-contours of Mandarin. The sustained field (SF) evoked by natural speech signals was significantly larger for Chinese than for German listeners. In contrast, the SF elicited by a horn tone was not significantly different between groups. Furthermore, the SF of Chinese subjects was larger when evoked by meaningful syllables compared to meaningless ones, but there was no significant difference regarding whether vowels were part of the Chinese phonological system or not. Moreover, the N100m gave subtle but clear evidence that for Chinese listeners other factors than purely physical properties play a role in processing meaningful signals. These findings show that the N100 and the SF generated in Heschl’s gyrus are influenced by language experience, which suggests that AC activity related to specific pitch contours of vowels is influenced in a top-down fashion by higher, language related areas. Such interactions are in line with anatomical findings and neuroimaging data, as well as with the dual-stream model of language of Hickok and Poeppel that highlights the close and reciprocal interaction between superior temporal gyrus and sulcus.

## Introduction

While in most Indo-European languages such as English and German pitch is used to transmit prosodic information, varying pitch contours are used in tonal languages such as Chinese at a syllable level to discriminate lexical meaning [1–3]. The lexical significance of pitch contours is a special challenge in speech processing for speakers of a non-tonal language. It is therefore an interesting question on which level neural processing differs between these groups.

Although neurons at lower levels, such as in the cochlear nucleus and the inferior colliculus, are thought to reflect the acoustic structure with extremely high fidelity [4], current studies on frequency following responses (FFR) suggest that these stations are not merely passive relays during the transmission from periphery to higher stages along the auditory pathway. Evidence for a selective behavior of the primary auditory cortex (AI) is provided by neurophysiological recordings where a group of AI neurons in the squirrel monkey responded selectively to species-specific calls [5]. This finding was corroborated by Wang et al. [6] who registered sustained responses elicited by particular sounds in primary auditory cortex and lateral belt areas. Thus, neurons in AC and higher stages seem to represent abstract auditory entities [7].

Non-invasive neurophysiological techniques, such as EEG and MEG, can be employed to register pitch related neural activity at different processing stages. FFR recordings catch sustained phase-locked activity in the rostral brainstem up to 1000 Hz, thus reflecting temporal and spectral features of speech sounds with extremely high fidelity [8, 9] comprises an explicit representation of pitch [10,11]. Candidate regions for the specific representation of pitch related information have been found at the cortical level. Multiple imaging techniques like positron imaging (PET) [12], functional magnetic resonance (fMRI) [13], magnetoencephalography (MEG) [14, 15], as well invasive corticographic and depth electrode recording [16] identified the antero-lateral end of Heschl’s gyrus (HG) in both hemispheres as an area where a transformation to pitch related activity is performed. This ‘pitch center’ has also been identified in recordings of non-human primates [17]. However, in comparison to the brainstem, phase-locking properties of cortical neurons are limited to sub-pitch rates, which suggests that different coding properties are employed at both levels. Furthermore, in comparison to the core region (AI), neurons in this area are expected to respond with longer latencies and greater specificity [18, 19]. Further adjacent areas to the core region comprise the planum temporale (PT) located posterior to HG, the antero-lateral planum polare and, lateral to superior temporal gyrus (STG), the superior temporal sulcus (STS) that is thought to be involved in phonetic and phonological processing, even during passive listening conditions [20, 21]. An important principle of these regions is their reciprocal organization, including a massive top-down stream [10, 18, 19].

MEG recordings, in conjunction with source analysis, are a practical and powerful way to investigate the time course of selective cortical areas with high temporal resolution [22]. Transitions from non-regular to regular sounds result in the pitch onset response (POR), a prominent negative deflection that is highly correlated with pitch salience [23]. Longer period sounds like vowels, musical and non-musical sounds evoke a negative sustained field (SF) which builds up over about 400 ms after tone onset and lasts until tone offset [24, 25, 26]. Dipole model localization techniques with multiple generators in each hemisphere show that pitch specific SF sources are located in, or close to, the pitch center [14]. In contrast, isolated intensity changes of regular and irregular sounds lead to correlated magnitude changes of a separate SF source located posterior to the pitch center, in PT. This separation of the pitch center was validated using imaging and intracranial techniques [12, 13, 16, 27].

Evidence that the representation of language related periodic sounds at several stages of the auditory pathway is altered by long-term experience as well as extensive training comes from a series of recent electrophysiological experiments. FFR responses of Chinese listeners were enhanced in reaction to Mandarin specific pitch contours, which shows that language experience influences auditory processing at stages as low as the brainstem [28, 29, 30]. Interestingly, such effects were not recorded for pitch contours that are not part of the native language. Therefore, it is assumed that such enhancements are experience dependent and do not reflect a superior periodicity representation *per se*. Further experiments using non-speech Mandarin tone contour homologues provided evidence that such a specific enhancement is also represented in auditory cortex.

In order to study the difference in pitch processing of Chinese and English speakers at the cortical level, Krishnan et al. [31] employed noise- *f*_0_-contour transitions to investigate the AEP evoked by the onsets and the equivalent of Mandarin tone T2. Detailed analyses showed that the immediate POR was of comparable morphology for all contour conditions, whereas *f*_0_-dynamics resulted in series of specific peak-to-peak relationships, depending on the *f*_0_-contour. While Chinese listeners showed a larger POR than the English participants, no differences were found for the subsequent pitch response elicited by the *f*_0_-modulation. The FFR, which were recorded in the same session, revealed a similar pattern, i.e., larger magnitudes for regular sounds, which is in line with several earlier observations (for a summary, see [31]). Such enhancements are regarded as the result of adaptive processes of pitch related mechanisms which are modulated by extrasensory high-level processes, i.e., stronger FFR representations are seen as a result due to corticofugal effects.

Thus, the stages from IC to AC act as a wide spanning feedforward and feedback network which adapts in order to provide optimal representations of behaviorally relevant stimuli. Such domain specific plasticity effects have also been observed in musicians. Pantev et al. [32] showed that musicians exhibited larger N1 responses for piano tones, but not for carefully matched pure tones, which may also be interpreted as a use-dependent reorganization in the auditory cortex (see also [33]).

In contrast to the large amount of studies that focused on transient responses elicited by speech sounds, only few experiments on the sustained potential (SP) and field (SF) evoked by vowels provided evidence that sustained activity might be important to assess language specific effects. Early investigations revealed that synthetic vowels with distinct formants and formant relationships (F1-F2 distance) yield different activations when compared to tones or single formants of equal loudness [24, 25]. This was interpreted as the result of interactions at one or multiple stages in AC and surrounding areas. Further, the comparison of vowel series and carefully matched noise bursts in continuous stimulation paradigms revealed stronger transient and sustained responses to vowels than to noise bursts [34]. Interestingly, the generators of the vowel related responses were found to reside in anterior regions of AC, while noise related N1 and SF responses were mapped onto posterior sites. Similar spatial separations and amplitude differences of periodic in contrast to aperiodic speech sounds were revealed using whole head MEG [26]. A recent combined MEG/EEG study showed that the specific activity elicited by synthetic vowels closely matched non-linguistic regular sounds [35]. Moreover, the generators were located within the same area along the antero-lateral HG, adjacent to the primary auditory cortex. These findings suggest that auditory processing passes through some kind of filter; however, it remains unclear which factors actually shape this filter, and when, how, and to what extent it is activated.

**Fig 1:**
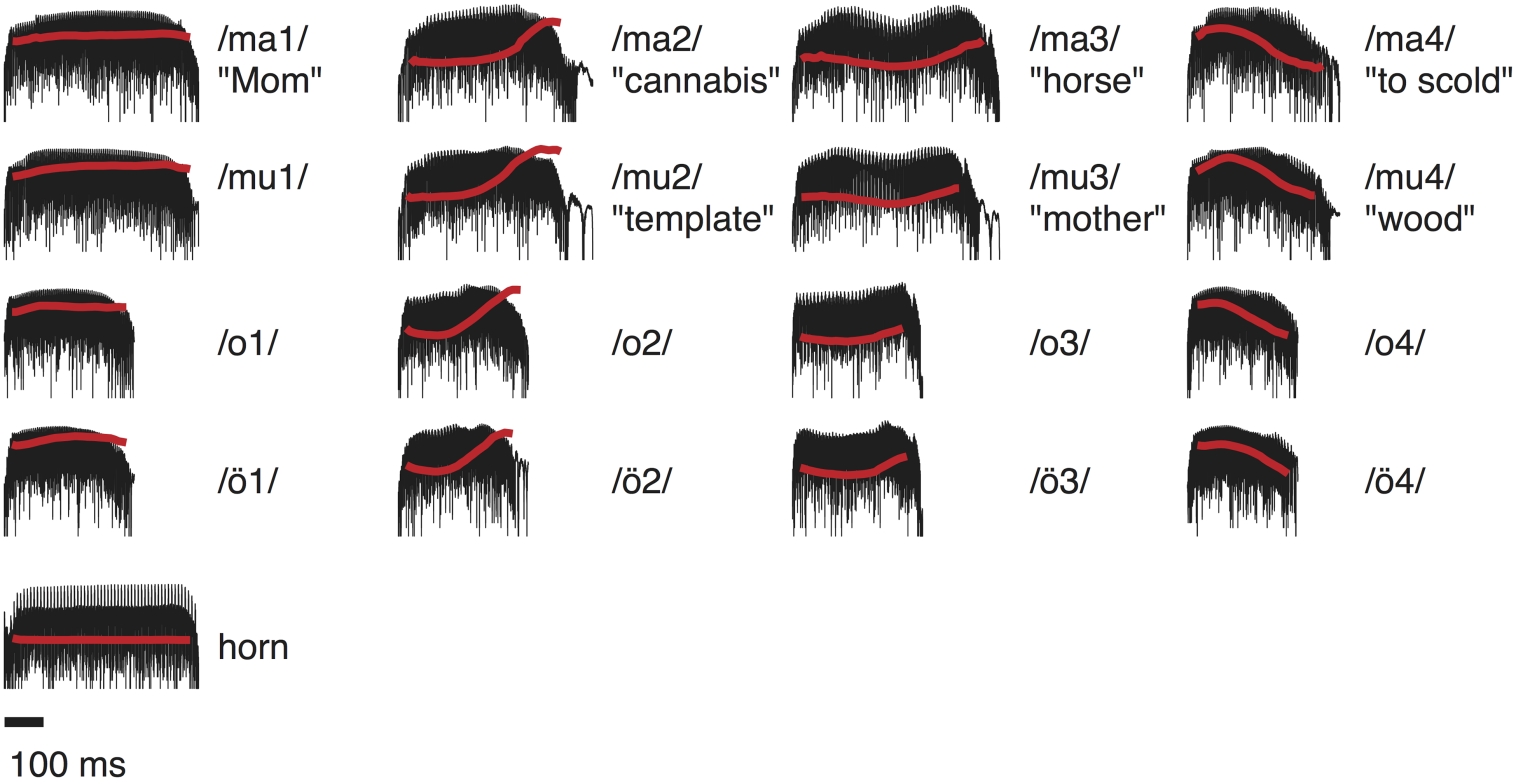
Stimuli. Squared sound pressure curves (black) of the vowels and syllables recorded from a native speaker, as well as a French horn tone. The red line indicates the pitch contour (*f*_0_) for each sound.

In this context, we addressed the question of language-specific effects during auditory processing in terms of two research questions: First, do cortical responses differ with respect to stimuli that show language-specific features on phonological and semantic dimensions? For this research question, we used the vowels <o> / <ö> and the syllables <ma> / <mu> as stimuli. These sounds exhibited the typical pitch contours according to the four tones in Mandarin (Fig 1), which is not a feature of the German phonological system. We chose <o> and <ö> because both vowels exist in German while only <o> exists in Chinese. The syllables <ma> and <mu> were employed because this allowed us to contrast meaningful and meaningless syllables in Chinese. Second, in order to investigate differences in linguistic and non-linguistic processing, we compared the cortical responses to a natural, French horn tone with the activity in response to a fixed pitch and the spoken syllable <ma1>. Based on the results of this study, we propose that the mother tongue has a major impact on auditory processing at early stages of the auditory cortex. The analyses showed that the SF of Chinese subjects was larger than the SF of German listeners when stimuli consisted of natural speech sounds. Furthermore, the detailed analysis of the Chinese listeners revealed that meaningful syllables evoked larger sustained responses as compared to meaningless ones. The idea that these observations mirror language related effects was further supported by the result of the second experiment, where the SF evoked by a horn note exhibited comparable amplitudes between groups, while the group difference for the syllable <ma1> reflected the contrast between both groups of experiment 1.

## Materials and methods

### Subjects

40 adult listeners (20 native speakers of Mandarin Chinese, 18 females and 2 males; 20 native German speakers, 8 females and 12 males), with no reported history of peripheral or central hearing disorders, participated in experiment 1. The mean age (± standard deviation) was 24 ± 3 years for the Chinese listeners, and 26 ± 4 years for the German listeners. All subjects, except one German listener, were right handed. Chinese listeners were born and grew up in the homeland of China. They had regular English lessons from the 3^rd^ grade on. Since they studied German as a foreign language at Heidelberg University, they also had German lessons in their homeland. However, we took care that, at the time of MEG recordings, the participants had been in Germany for less than six months. German listeners started English courses from the 5^th^ class on. Further data regarding musical experience were not assessed.

A subgroup of the Chinese (1 male, 10 female) and German listeners (7 males, 9 females) participated in a second experiment to investigate the difference between musical and phonetic stimuli. All subjects participated after providing written informed consent. The study protocol was in accordance with the declaration of Helsinki and was approved by the ethics committee of the Heidelberg University’s Medical School.

### Stimuli

Fig 1 depicts the squared sound pressure curves and pitch contours of the stimuli. Pitch contours were extracted using the Praat software, version 5.3.44 [36]. All speech signals were recorded from an adult male native speaker and showed the pitch contour of the four Mandarin tones as shown in Fig 1. The tones were clearly recognized by all native Chinese speakers. The vowel <o> exists as a phoneme in both languages, whereas <ö> (o-umlaut) does not exist in Mandarin. The syllable <ma> is meaningful for all four contours, but <mu> is meaningful only for the three tones <mu2><mu3><mu4>. In contrast, the syllable <mu1> is meaningless.

In experiment 1, stimuli were presented in two different MEG recording sessions, which lasted 24 and 28 minutes, respectively: (1a) vowels <o1>, <o2>, <o3>, <o4>, <ö1>, <ö2>, <ö3>, <ö4>, (1b) syllables <ma1>, <ma2>, <ma3>, <ma4>, <mu1>, <mu2>, <mu3>, <mu4>. Experiment 2 consisted of a single 7-minutes session with the aim of contrasting the syllable <ma1>, which has a flat pitch contour, with a French horn tone (b-flat, 117 Hz) from a database [37]. The duration of this musical tone was matched to that of the syllable <ma1> (see Fig 1). Vowels and syllables were recorded using a Brühl & Kjaer microphone, Type 4193, connected to a Brühl & Kjaer preamplifier, and a mixing desk Mäckie, Type 1402-VLZ Pro. A/D conversion and recording was carried out using an Audio Interface (RME Hammerfall DSP Multiface), a Dell Latitude D830, and the software Audacity 2.0.2. [38]. Stimuli were presented diotically to the listeners via Etymotic Research (ER3) earphones with 90 cm plastic tubes, equipped with foam ear pieces. The effective bandwidth of this setup is about 85–2380 Hz. Thus, all *f*_0_ modulations as well as the 2^nd^ and 3^rd^ formant were within the audible range. The overall stimulation level was set to 72 dB SPL using a Brüel and Kjær measuring amplifier (Type 2610) and an ear simulator (Type 4157). The stimuli were played at 48,000-Hz sampling rate using a 24-bit sound card (RME ADI 8DS AD/DA interface), an attenuator (Tucker-Davis Technologies PA-5) and a headphone buffer (Tucker-Davis Technologies HB-7). The order of runs was randomized, as well as the order of stimuli within each run. Each stimulus was presented 180 times, and the stimuli were separated by an inter-stimulus-interval of 700 ms. Randomization ensured that no meaningful sentences or expectations were built up.

### MEG recording

The gradients of the magnetic fields were recorded using a Neuromag-122 whole-head MEG (Elekta Neuromag Oy, Helsinki, Finland) inside a magnetically shielded room (Imedco, Hägendorf, Switzerland). Data were low-pass filtered (330 Hz) and sampled at a digitization rate of 1000 Hz. Prior to recordings, the nasion and two pre-auricular points, as well as 32 surface points, were digitized using the Polhemus 3D-Space Isotrack. During the MEG recordings, subjects watched a silent movie (with subtitles) of their own choice, and listened passively to the stimuli. Cortical responses were averaged for each stimulus using the BESA program 5.2 (BESA Software, Gräfelfing, Germany). Epochs (-300 to 1000 ms) exceeding amplitudes of 8000 fT/cm, or gradients of 800 ft/cm/ms, were not included in this procedure. On average, 167 sweeps per subject and condition remained for analysis in the Chinese group, and 175 sweeps in the German group. The baseline was computed by averaging the data from -100 to 0 ms, relative to stimulus onset.

AEF were analyzed in two ways: (i) for model independent information of the total activity elicited by a stimulus irrespective of localization, we computed the root mean square (RMS) of all channels, separately for each condition and each subject. A linear regression was employed to remove drift artifacts. (ii) In order to obtain specific information regarding the source localization and specific activation within the auditory cortex, we employed a spatio-temporal source model [22] with one equivalent dipole per hemisphere. This analysis was based on a spherical head model and a homogeneous volume conductor. The radius and position of the spherical model was derived from the individual digitized head surface points. A spherical MEG model allows to derive a sufficient representation of generators of cortical sources located in the temporal lobe [39]. Dipole fits were derived for the N100m as well as for the SF, and were performed separately for each stimulus condition and subject. The N100m was fitted around the peak covering the interval from baseline to baseline. The fit of the SF was based on the time interval ranging from 300 to 600 ms after stimulus onset, which included the most prominent sustained portion of the averaged waveform. All fits were based on unfiltered data. Data analysis was carried out using Matlab 7.11 (The MathWorks, Nattick, MA, USA).

### Statistics

Table 1 depicts all data as means ± standard errors. Statistical analysis was carried out using ANOVA, with the independent factor *group* and the within subject factors *meaningful/meaningless* and *hemisphere*, in order to test main and interaction effects. *η*^2^values are provided to assess the effect size of significant effects.

**Table 1:** AEF mean values and standard errors of the Chinese and German listeners. Values were obtained from RMS, averaged across all gradiometers (RMS), and from a fit with one equivalent dipole in the left and right hemisphere. ISF represents the sustained field integrated from 300 ms to 1000 ms after tone onset. **Rows 1a-d**: average values of all phonetic stimuli used in experiment 1 for Chinese and German listeners. **2a-e**: averages across all stimuli for the second experiment which used the musical tone b-flat of a French horn, and the spoken syllable <ma1>. **3a-b**: averages across all four notes of the vowels <o> and <ö>. Only <o> is part of the Chinese phonetic system. **3c**: difference between the specific responses evoked by <o> and <ö>. Signals of the left and right dipole waveforms were averaged since there was no indication of lateralization. **4a-c**: Average values of the responses evoked by the meaningful tones <ma> and the meaningless tones <mu> as well as the difference between these two classes. **5a-f**: amplitudes of the transient N100m responses averaged across all stimuli and hemispheres (5a) as well as for both hemispheres separately (5b and c) and the difference between hemispheres. **5e-f** represent average values across all meaningful and meaningless of Chinese and German listeners (all vowels and the syllable <mu1>; “Meaningful” denotes the average across all stimuli being meaningful for Chines listeners (all tones of <ma> and <mu2>—<mu4>)).

		Chinese		Germans	
	Stimuli	Dipole, ISF	RMS, ISF	Dipole, ISF	RMS, ISF
		[nAm*s]	[fT s/m]	[nAm*s]	[fT s/m]
1a	All Phon. Stim.Both hemisph.	-9.1±1.02	3.62±0.38	-6.13±1.00	2.19±0.25
1b	Left	-8.84±1.09		-6.19±1.18	
1c	Right	-9.36±1.06		-6.06±0.88	
1d	Left-Right	0.52±0.70		-0.13±0.61	
2a	horn	-17.26±2.60	6.74±0.62	-15.61±2.04	5.73±0.71
2b	Horn, left	-16.3±2.50		-14.67±2.18	
2c	Horn, right	-18.22±2.72		-16.54±2.60	
2d	/ma1/*	-13.28±0.64	5.38±0.40	-9.32±1.42	3.73±0.45
2e	horn-/ma1/*	-3.98±2.59	1.35±0.61	-6.84±1.63	2.00±0.65
3a	/o/	-6.83±1.08	2.53±0.31	-4.91±1.00	1.84±0.23
3b	/ö/	-7.34±1.12	3.03±0.32	-5.2±1.01	1.71±0.21
3c	/o/-/ö/	0.51±0.50	-0.49±0.26	0.29±0.41	0.13±0.90
4a	/ma1/	-13.44±1.29	4.98±0.71	-8.92±0.98	3.32±0.38
4b	/mu1/	-10.46±0.85	4.47±4.47	-8.70±1.43	3.05±0.42
4c	/ma1/-/mu1/	-2.98±0.89	0.51±0.49	-0.22±1.05	0.28±0.39
		**Dipole, N100m**	**RMS, N100m**	**Dipole, N100m**	**RMS, N100m**
		[nAm]	[fT/m]	[nAm]	[ft/m]
5a	All Phon. stim, Both hemisph.	-17.64±2.16	15.41±1.41	-16.05±2.39	13.60±6.30
5b	left	-17.26±2.33		-14.89±2.67	
5c	right	-18.02±2.35		-17.22±2.42	
5d	left-right	0.76±1.82		2.33±1.77	
5e	meaningless	-18.08±2.38	15.80±1.50	-18.28±2.55	14.23±1.04
5f	meaningful	-17.07±2.04	15.20±1.48	-13.19±2.36	12.80±0.91

## Results

### Global effects of speech stimuli

**Fig 2:**
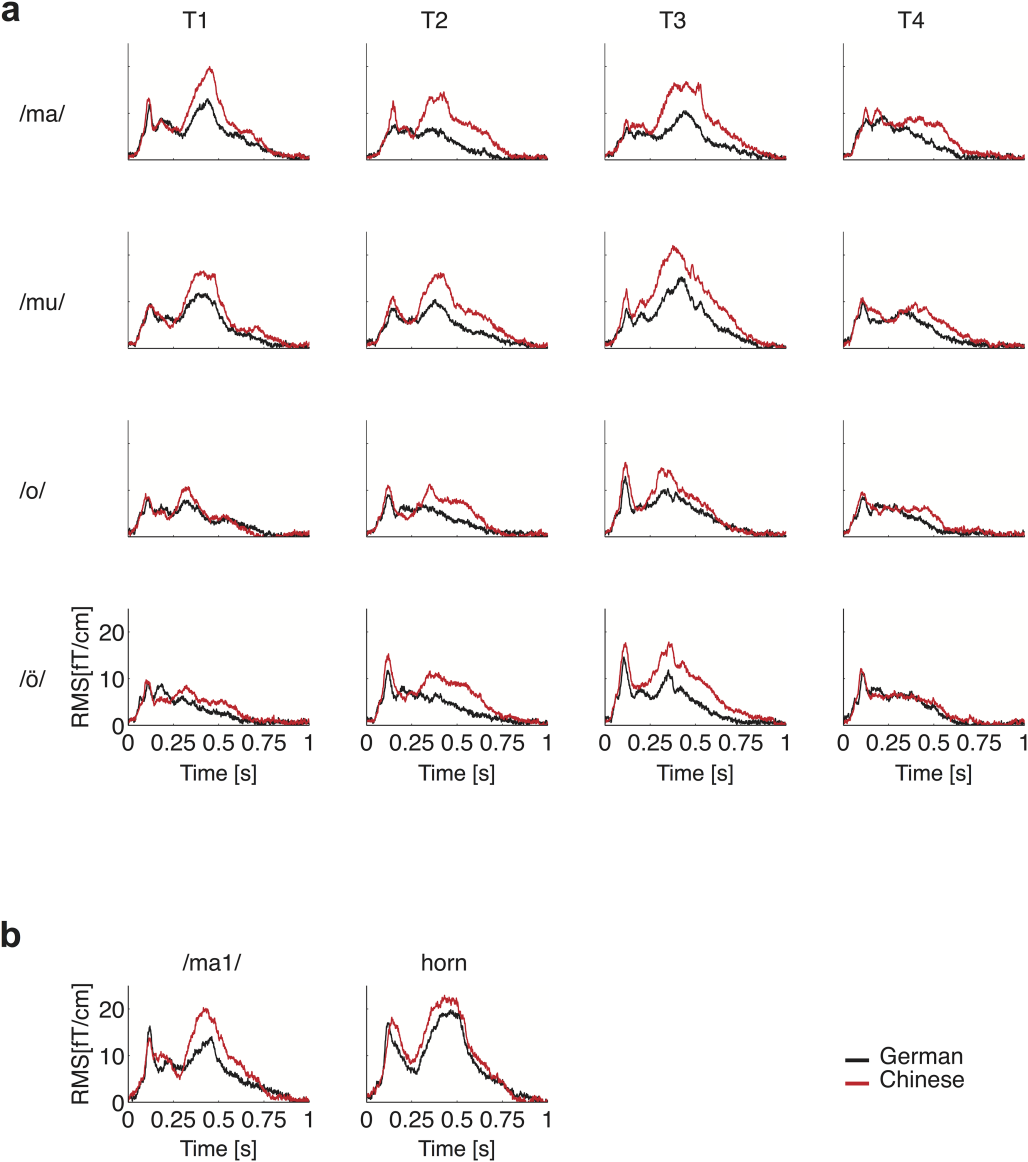
Grand-average auditory evoked fields. **(a)** RMS magnetic field gradients (based on all gradiometers) of all syllables and vowels and pitch contours (T1-T4) used in experiment 1 (red: *n* = 20 Chinese listeners; black: *n* = 20 German listeners). **(b)** Grand-average RMS evoked by the syllable <ma> and the horn tone of experiment 2 (red: *n* = 11 Chinese listeners and black: *n* = 14 German listeners).

Fig 2 shows the model independent RMS data of the magnetic field gradient for all stimuli. The AEF morphology clearly exhibited prominent deflections at about 50,100, and 200 ms after stimulus onset, corresponding to the P50m, N100m and P200m, and followed by the SF with a maximum amplitude around 400 ms after stimulus onset. The SF decrease of the temporal waveform after 400 ms is due to the fact that syllables and vowels did not exceed a duration of 330–490 ms. Table 1 shows the results for the temporal integral of the SF, referred to as ISF. The lower integration limit is set to 300 ms after stimulus onset, where a temporal overlap with the transient components (P50m, N100m, P200m) can be ruled out. The upper limit is 1000 ms after stimulus onset when the signal has reached baseline.

**Fig 3:**
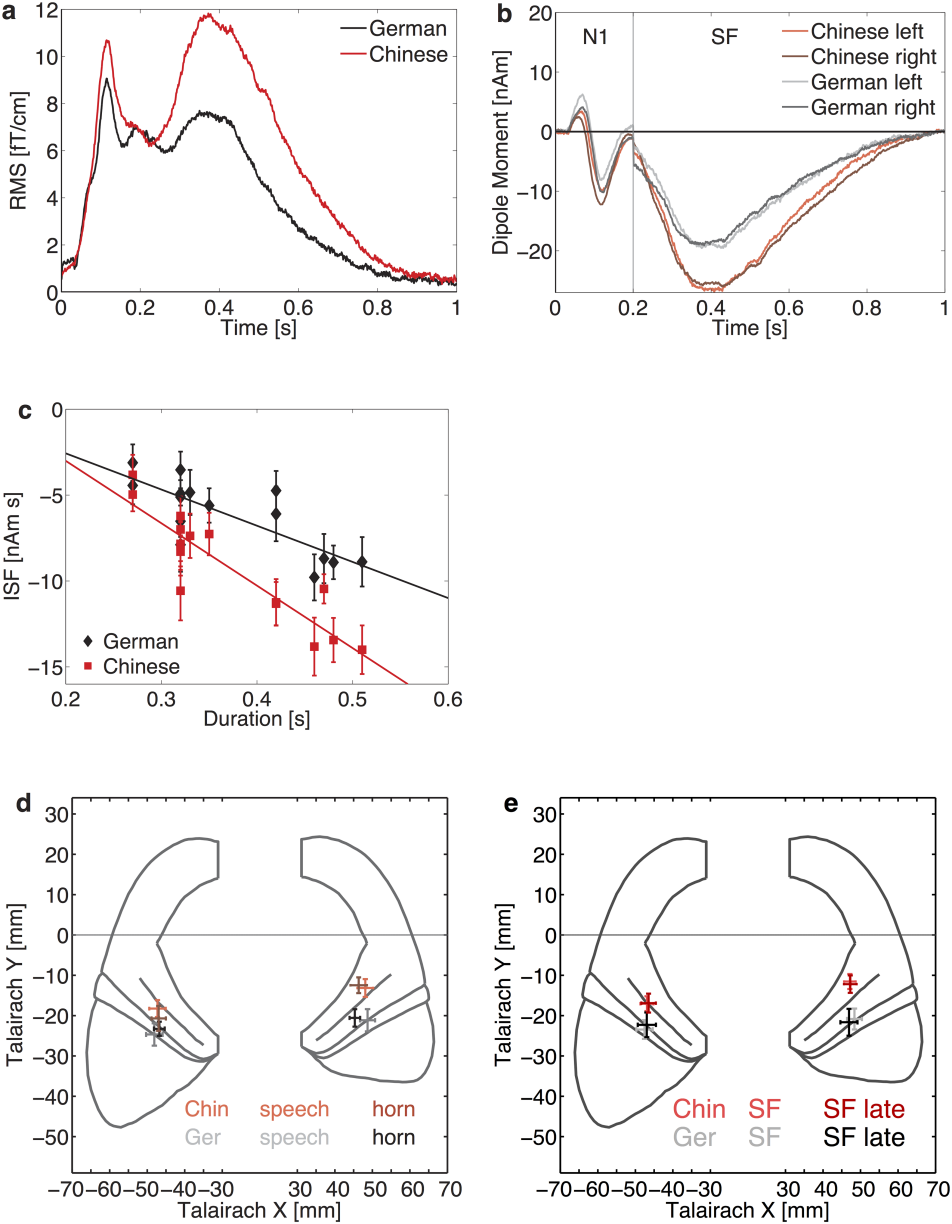
Grand-mean waveforms. **(a)** Grand-mean RMS of all pooled phonetic responses used in experiment 1, for Chinese (red) and German (black) listeners. **(b)** Grand-average source waveforms of all pooled phonetic responses of the N100m and the SF with one equivalent dipole in each hemisphere. Waveforms shown in the time interval from 0–200 ms were based on a fit of the transient N100m. The waveforms shown in the second part of the plot (200–1000 ms) were derived from a dipole model of the SF which was based on a fit interval from 300–600 ms after tone onset. Note the similarity of data derived from the left and right hemisphere. **(c)** Integrated sustained field (ISF) plotted against stimulus duration. The error bars depict the standard error of the mean. **(d)** Projection of the mean Talairach coordinates of the dipole models of both groups for the speech sounds and the horn tone onto the map of Schneider et al. [40]. The error bars represent the standard errors of the mean along the *x*- and *y*-axis. **(e)** Mean Talairach coordinates of the ISF-generators for speech signals, derived from the standard fit interval (300–600 ms after tone onset) of the Chinese (red) and German (grey) listeners and the corresponding positions based on the late fit interval (500–800 ms, Chinese (dark red) and German listeners (black)).

The main result of our investigation is the significant group difference of the ISF for the spoken signals shown in Figs 2, 3a and 3b, and Table 1. The model independent RMS, integrated over the time interval from 300 to 1000 ms after stimulus onset, was by a factor of about 1.5 larger in the Chinese group than in the German group. The difference was Δ = 1.43 ± 0.45 fTs/cm, see Fig 3a and Table 1, line 1a, (RMS—*group*: *F*(1,38) = 10.53, *P* = 0.025, *η*^2^ = 0.217; *meaning*: *F*(1,38) = 47.17, *P*<0.0001, *η*^2^ = 0.55; group*meaning: *F*(1,38) = 8.66, *P* = 0.0055, *η*^2^ = 0.19). The average current-dipole strength depicted in Fig 3b showed the same pattern, the difference was Δ = 3.0 ± 1.4 nAm s, see Table 1, line 1a (*group*: *F*(1,38) = 4.72, *P* = 0.0362, *η*^2^ = 0.11). Furthermore, we observed a large main effect of *meaning* (*F*(1,38) = 39.81, *P*<0.0001, *η*^2^ = 0.51) which is probably due to the length of the stimuli. However, the group*meaning interaction indicated a specific enhancement in Chinese listeners (*F*(1,38) = 7.11, *P* = 0.0112, *η*^2^ = 0.16), while no hemispheric effects could be observed (*hemisphere*: *F*(1,38) = 0.16, n.s.; group*hemisphere: *F*(1,38) = 0.52, n.s.).

Detailed analysis of the SF revealed a strong correlation between the duration of the stimulus and the integrated response (Chinese group: *r* = 0.91, *P*<0.0001; German group: *r* = 0.79, *P* = 0.001). In Fig 3c, the ISF of the phonetic stimuli is plotted against stimulus duration. The extrapolated thresholds of the regression lines agree within the errors for both groups, but for the Chinese group, the slope has nearly twice the value of the German group's slope.

A significant group-specific difference was also observed for the average position of the SF generators, as obtained with a spherical head model and without adjustment to individual anatomy (see Fig 3d**)**. The dipole position in the Chinese subjects was on average by 0.72 ± 0.23 cm more anterior in comparison to the German listeners (*t*(38) = 4.1, *P* = 0.0002). These results are based on the SF dipole fit from 300–600 ms after stimulus onset. In order to ensure that the SF is based on the same generator over the whole time interval, and not related to the N400 which is typically elicited by semantic effects, we reanalyzed the SF fit using a second fit, based on the time interval from 500–800 ms after stimulus onset. As can be seen in Fig 3e, the position of the sustained field based on the additional fit coincides within an acceptable error margin with that of the standard fit from the interval from 300 to 600 ms after stimulus onset.

### Natural speech vs. musical stimuli

Since we were generally studying language related effects in the signals at the cortical level, we investigated, in a second experiment, the spoken syllable <ma1> (which as the first Mandarin tone exhibits a rather fixed pitch contour) in contrast to a (not language related) French horn tone with a fixed pitch of *f*_0_ = 117 Hz (b flat). Both tones were presented in random order to a sample of 11 Chinese and 16 German listeners who also participated in the first experiment (see Fig 2b and Table 1, lines 2a-e). In contrast to the speech stimuli, the ISF evoked by the French horn tone did not differ significantly between groups (RMS /ma1/—horn: *group*: *F*(1,25) = 2.94; n.s.; *stimulus*: *F*(1,25) = 10.02, *P* = 0.004, *η*^2^ = 0.29, group*stimulus: *F*(1,25) = 0.38, n.s.; dipole analysis: *group*: *F*(1,25) = 1.42, n.s.; *stimulus*: *F*(1,25) = 13.83, *P* = 0.001, *η*^2^ = 0.36; group*stimulus: *F*(1,25) = 0.97, n.s.). Post-hoc tests showed no difference for the French horn (RMS—*group*: *F*(1,25) = 0.80, n.s.; dipole analysis–*group*: *F*(1,25) = 0.12, n.s.; *hemisphere*: *F*(1,25) = 8.61, *η*^2^ = 0.26; group*hemisphere: *F*(1,25) = 0.24, n.s.). However, in line with the results of Experiment 1, we observed a highly significant group difference for signals evoked by the syllable <ma1> in the post-hoc tests (RMS: post-hoc test /ma1/: *F*(1,25) = 5.35, *P* = 0.0292, *η*^2^ = 0.15 *t*(25) = 2.31, *P* = 0.029; dipole analysis: *group*: *F*(1,25) = 4.86, *P* = 0.0369, *η*^2^ = 0.16; *hemisphere*: *F*(1,25) = 5.30, *P* = 0.030, *η*^2^ = 0.17; group*hemisphere: *F*(1,25) = 1.67, n.s.).

### Phonological effects

**Fig 4:**
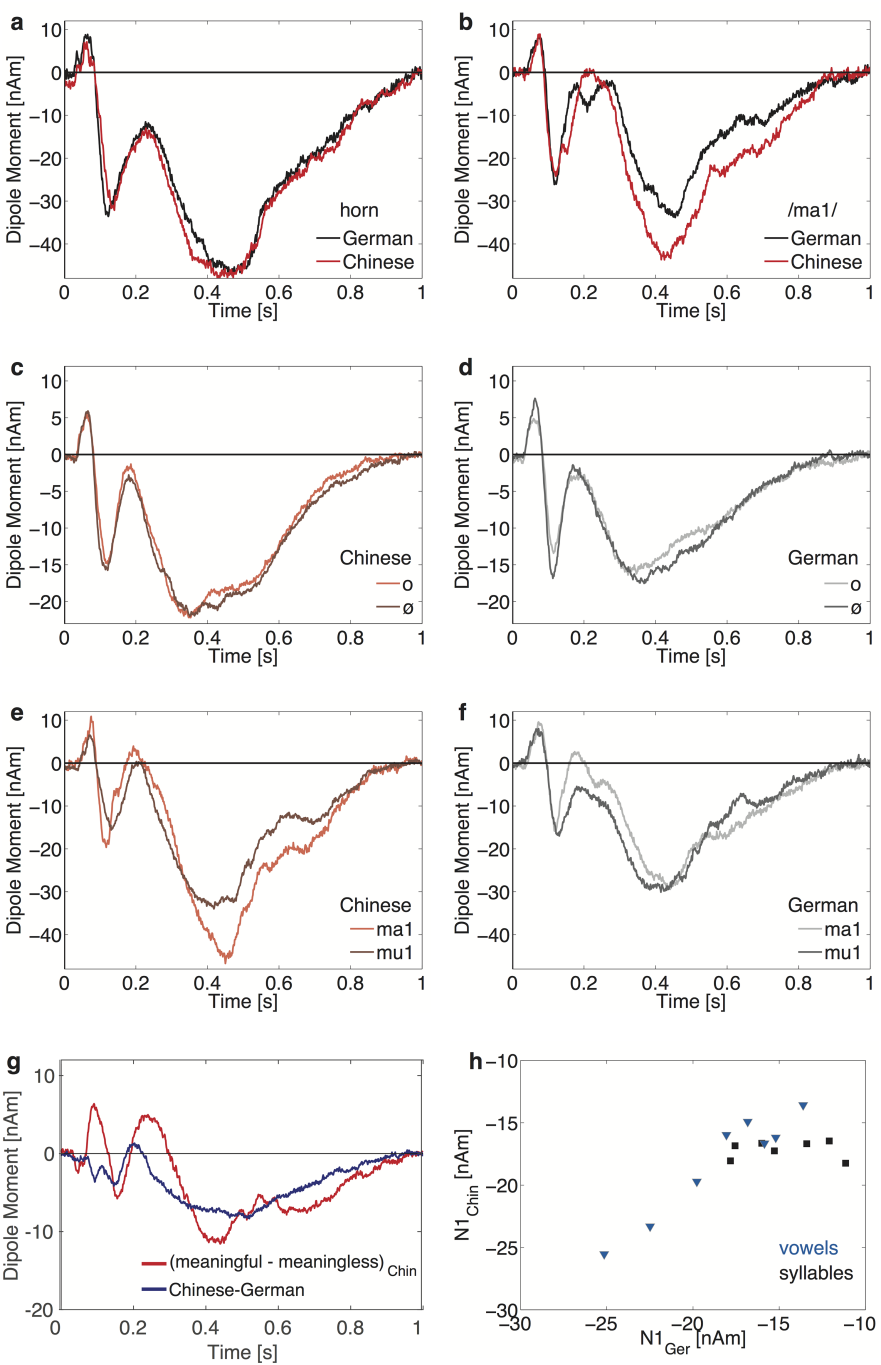
Grand-average source waveforms for specific stimuli. **(a)** Responses pooled across hemispheres to a French horn tone (b-flat 117 Hz) and **(b)** to the spoken stimulus <ma1> presented in experiment 2 to the Chinese and German listeners. Note the similarity of the AEF between groups for the horn tone in contrast to the significant larger response evoked by the syllable <ma> for Chinese listeners. **(c)** and **(d)** depict the grand-average source waveforms elicited by the vowels <o> and <ö> (averaged across all four tone conditions, T1-T4). While the vowel <o> occurs in both languages, <ö> is only part of the German language. **(e)** Responses to the syllables <ma1> and <mu1> of the Chinese group and **(f)** the German group. <mu1> has no meaning in Chinese, in contrast to <ma1>; in German, both syllables are meaningless. **(g)** Time-variant difference of the Chinese responses to the stimuli meaningful and meaningless phonemes (red curve), and the difference of the average responses to the all spoken stimuli between Chinese and Germans (blue curve). **(h)** N100m signals of the Chinese vs the German listeners for the meaningful syllables (black boxes: <ma1> -<ma4>, <mu2>-<mu4>), and the meaningless signals (gray triangles: all vowels and <mu1>). The Pearson correlation coefficient corresponding to the mean values of the meaningless signals (blue triangles) between groups is *r* = 0.96, (*P*<0.0001); however, no significant correlation was found for the meaningful syllables (black squares).

Whereas the vowel <o> exists in Chinese, the vowel <ö> does not. Fig 4c–4f show the comparison of the grand mean source waveforms for the Chinese and German subjects. Waveforms amplitudes did not show any effect concerning lateralization (*hemisphere*: *F*(1,38) = 0.59, n.s.; group*hemisphere: *F*(1,38) = 0.31, n.s.). The ANOVA of the ISF extracted from RMS and dipole analysis which included /o/-/ö/ as the factor *vowel*, and the four tones as the within-factor *contour*, revealed no difference between groups for the dipole solution (*F*(1,38) = 1.94, n.s.). However, the model free RMS data indicated a difference between groups (*F*(1,38 = 5.90, *p* = 0.02, *η*^2^ = 0.13)). Both approaches were not significant for vowel effects /o/ vs /ö/ (RMS: *F*(1,38), n.s.; dipole-solution: *vowel*: *F*(1,38) = 1.52, n.s.), however a significant interaction was found for the RMS data group*vowel (F(1,38) = 4.17, *p* = 0.0481) that failed to reach significance for the dipole solution (*F*(1,38) = 0.12, n.s.). Contour effects influenced the ISF (RMS: *F*(1,38) = 26.78, *p*<0.0001), *η*^2^ = 0.41; group*contour: *F*(1,38) = 3.65, *P* = 0.0225, *η*^2^ = 0.087; dipole solution: *contour*: F(1,38) = 17.78, *P*<0.0001, *η*^2^ = 0.32; group*contour: *F*(1,38) = 2.96, *P* = 0.0354, *η*^2^ = 0.072).

### Semantic effects

Furthermore, our paradigm included the comparison between the meaningful <ma1> and the meaningless syllable <mu1>. Fig 4e and 4f show the SF evoked by these stimuli for both groups (see also Table 1, lines 4a-c). In the Chinese group, the ISF evoked by the meaningful syllable <ma1> was significantly larger than that of the meaningless syllable <mu1> (*vowel*: *F*(1,19) = 11.25, *P* = 0.0033, *η*^2^ = 0.37). No difference was observed for the other minimal pairs where both stimuli carry meaning (<ma2> vs <mu2>: *F*(1,19) = 0.0, n.s.; <ma3> vs <mu3>: *F*(1,19) = 0.02, n.s.; <ma4> vs <mu4>: *F*(1,19) = 0.02, n.s.). As expected, no significant difference for the German group was observed (<ma1> vs <mu1>: *F*(1,19) = 0.05, n.s.; <ma2> vs <mu2>: *F*(1,19) = 1.07, n.s.; <ma3> vs <mu3>: *F*(1,19) = 0.44, n.s.; <ma4> vs <mu4>: *F*(1,19) = 0.86, n.s.). Of our 16 spoken stimuli, 9 are meaningless (all 8 vowels and <mu1>), and 7 stimuli meaningful (all <ma> and tone 2 to 4 of <mu>). The stimuli are acoustically too different to draw conclusions from a within-group comparison. Nonetheless, we found a highly significant difference between Chinese and Germans in the ISF for the meaningful stimuli (*F*(1,38) = 7.35, *P* = 0.01, *η*^2^ = 0.16), whereas the difference for the meaningless stimuli was not significant (*F*(1,38) = 1.97, n.s.), see Table 1, lines 5e-f. To illustrate the specific waveform morphology, Fig 4g depicts the difference between the signals evoked by <ma1> and <mu1> for Chinese listeners as a function of time. The distinction between the meaningless and the meaningful signal occurs only at about 400 ms after stimulus onset, whereas the group difference between Chinese and Germans is visible much earlier. The pattern is exactly the same for the difference between the responses to the averaged meaningful versus meaningless stimuli; also, the group difference sets in about 100 ms earlier than the difference to semantics. The large difference between the dipole moment evoked by <ma1> and <mu1> was partially due to a difference in the location of the effective generators; the difference was not so distinct for the RMS signal, where a significant difference was observed only in the time interval from 400 to 700 ms after tone onset (*t*(19) = 2.17, *P* = 0.042).

### Transient signals

The most prominent transient signal was the N100m. Waveforms and amplitudes of all signals are shown for both groups in Fig 3a and 3b, and Table 1, lines 5a-f. The N100m did not differ between groups (*F*(1,38) = 0.27, n.s.); furthermore, there was no general lateralization effect (*F*(1,38) = 1.43, n.s.) and no significant group*hemisphere interaction (*F*(1,38) = 0.27, n.s.).

There was, however, a subtle difference in the N100m responses for stimuli with different semantic aspects (meaning*group: *F*(1,38) = 3.85, *P* = 0.057). The N100m evoked by the stimuli that were meaningless for both the Chinese and the German group (the 8 vowels and <mu1>) correlated highly between these groups (*r* = 0.96, *P*<0.0001). In contrast, we observed no significant correlation between the groups for the N100m signals in response to the meaningful syllables <ma1>…<ma4> and <mu2>…<mu4> (*r* = 0.40, *P* = 0.1), see Fig 4h. The signal strength of the N100m for the <ma1> was larger than that for the <mu1>; but due to the large individual fluctuations, the difference (1.87 +/- 2.61) was far from significant. There was a highly significant mean amplitude difference (*t*(19) = 4.10, P = 0.0006) between N1m waves evoked by vowels and those evoked by the syllables for the Germans, but not for the Chinese, Table 1, lines 5e-f.

## Discussion

The aim of our investigation was to isolate language specific effects in cortical responses to acoustical stimuli, in Chinese listeners as compared to German listeners. An important difference between Mandarin Chinese and German is the use of pitch variations in a syllabic context, namely in the function of lexical tone for the discrimination of meaning [2, 3]. Beside this difference in the phonological systems, single syllables typically form words in Chinese, but not in German, and thus carry meaning. The main result of this cross-linguistic MEG study is a language related difference in the sustained response elicited by natural speech sounds. The current data suggest that phonological differences in the subjects’ mother tongue have a major impact upon processing in auditory cortex. The observed SF differences between Chinese and German listeners (Fig 3a–3b) extend previous investigations on sound processing at the subcortical level [41, 28, 42, 43, 30].

### Semantic effects

Our observation of language related contrasts of cortical sound processing poses the question as to the nature and level of language-specific influence factors. In Chinese listeners, the similarly strong SF to habituated and non-habituated vowels indicates a highly automated process that is sensitive to the physical properties of the speech stimulus. In an effort to assess how semantic differences influence the cortical response, we analyzed the activity to the meaningless <mu1> and the meaningful <ma1> sounds. Chinese subjects showed a significantly stronger SF for <ma1> than for <mu1> (see Fig 4e–4f). Although the lexical meaning of the other tones differ, no difference was observed for the other tone specific pairs, where *both* stimuli carry meaning. This effect can be expected, because the acoustical properties are very similar; moreover, due to randomization, no meaningful sentences were assembled during stimulus presentation. For similar reasons, one would not expect any significant difference for the German group, which indeed was the case in our study. This indicates that, in Chinese speakers, the difference results from the meaningfulness of a specific syllable-tone cluster, and not from pitch processing *per se* as a mere low-level (physical) characteristic of cortical auditory processing.

Further evidence for such a top-down contribution comes from the temporal structure of the SF. As can be seen from Fig 4g, the difference between the signals in response to meaningful and meaningless syllables occurs at 400 ms after onset, which is significantly later than the group-specific difference between Chinese and German listeners. This might reflect recent EEG and MEG recordings in passive listening experiments with random series of words, where meaningful words elicited larger responses, as compared to non-words [44–46].

Taken together, this pattern indicates that SF generators are influenced massively, and in an immediate trial-to-trial fashion, by higher cortical stages like STG and STS. This view is supported by various neuroanatomical and imaging data. Information processing takes place in a hierarchical manner from core to belt and para-belt regions [19]. Especially para-belt areas show massive connections with auditory subregions of STS, a region which is highly specialized for speech perception and language processing [47]. This finding is closely resembled in combined structural and resting state (f)MRI recordings that showed strong interconnections of STG, temporal, frontal and parietal regions with STS [48]. This pattern supports the dual-stream model of Hickok and Poeppel [49, 50, 51] according to which early speech processing stages, including the tight interaction between STG and STS, occur bilaterally. Empirical evidence for a *bilateral* activation also comes from a fMRI meta analysis in which a core region was identified in the temporal lobes anterior to HG [52].

Further important insights into the phonetic coding properties of the STG are given by recent high-densitiy electrode-grid recordings in human listeners [53]. High gamma oscillations in combination with cortical surface potentials showed a distributed spatiotemporal activity pattern when subjects listened to continuous speech spoken by hundreds of people. The analysis revealed a distinct, highly selective map of the complete English phonetic inventory within different STG sites. Furthermore, the detailed inspection of vowel parameters exhibited a strong representation of fundamental and formant variability in STG. Thus, it is plausible that specific STG representations interact instantaneously with generators that are located in adjacent anterior AC fields which are, in turn, driven by the regularity and salience of incoming sounds (25, 35). The output of this stage presumably contains abstract *f*_0_ and formant statistics, and it is conceivable that this output is fed directly into the feature maps of STG, as decribed by Mesgarani et al. [53]. First, such an interaction could explain the general finding that SF is enhanced when subjects listen to vowels and periodic sounds with distinct *f*_0_ statistics and specific formant relations, like the French horn. Second, such a mechanism could explain further findings concerning experience related differences in language and music, which were shown to be specific rather than generalized [32, 41, 31], in line with our data. We suggest that extensive musical training and language experience develop augmented stable feature maps in STG for specific *f*_0_ contours and formant relations, or for an instrument which is played several hours a day. Based on such maps, relevant information represented in lateral HG is extracted in order to sharpen features that are needed to provide valid and reliable representations regarding a syllable or a musical instrument. Distinct representations of that kind might pose an underlying principle for the develop of strong perceptual categories. This view is consistent with the finding that Chinese in contrast to English listeners exhibit more dichotomous psychometric functions as well as stronger neural coding for Mandarin tones [54].

Since the SF lasts until the end of a sound, it probably reflects lateral HG activity involved in a continuous feedback-feedforward process that also extends to subcortical regions, as suggested by morphological and physiological top-down routes from non-primary AC to inferior colliculus (IC) [9, 55]. The cortico-collicular system uses a direct projection originating in AC, bypasses thalamus and terminates in IC. While primary AC connections are tonotopically organized, further IC input stems from multiple areas within AC. In other words, AC modifies sensory processing in IC and acts as a gating or gain-control system that again influences the ascending input to AC; as a result, this improves stimulus coding. Since stages beyond IC exhibit a considerable amount of redundancy reduction [7, 56], and IC represents a stage where spectro-temporal information is represented with high-fidelity, the corticofugal system influences IC to adjust and update the ascending flow of sensory representation fast and on-line, when necessary. As a result, FFR representations are larger in Chinese participants when they listen to language related Mandarin tone contours [41]. At this point, it is unclear whether higher stages like STS and further fields are involved in the representation of meaningful and meaningless syllables in AC, as observed in our experiments. However, given the detailed and distinct representation found by Mesgarani et al. in STG, such feature maps could easily contain separate representations to map the different syllables of the four /ma/ and /mu/ tones. This could explain our specific SF effects which were found in passive listening conditions, even when subjects watched a silent movie with subtitles.

### Phonological effects

To further investigate the phonological status of the vowels in the linguistic systems of Chinese and German, we compared the AEF in response to <o> and <ö>, where the latter is not part of the Chinese vowel system. As expected, no difference was observed in the activation patterns of the German subjects, but there was also no difference in the neural responses of the Chinese speakers with respect to these two vowels. This result indicates that the between group SF difference regarding <o> and <ö> is not related to habituation to specific stimuli, but rather to the physical structure, reflecting the tonal contour of the stimulus. Although Chinese listeners are not habituated to the vowel <ö>, they are evidently sensitive to its temporal and structural properties, e.g., the rich harmonic spectrum of the formant and the dynamics that <ö> shares with other vowels. This observation is also in line with FFR results provided previously (for a summary see [31] and mirrors the above mentioned STG-AC interaction that in turn influences IC. To prove this suggestion, future experiments should employ non-native *f*_0_-contours.

### Musical versus linguistic stimuli

We investigated neural signals that were evoked by linguistic versus musical stimuli. In contrast to the SF amplitude enhancement elicited by vowels and syllables in Chinese subjects, the French horn stimulus in Experiment 2 led to a SF of comparable magnitude in both, Chinese and German listeners while group differences were found for /ma1/. This result pattern is again consistent with the above described model assumptions, and it might be viewed as the result of some language- or speech-related filter characteristic that is closely associated with the dynamic changes in multiple speech sound parameters like pitch contours [44, 4].

The similar cortical response to the horn note between groups does not match the findings of Bidelman et al. [57] who observed cross domain effects of music and language at the level of the brainstem. However, the relation between FFR and SF is largely unknown. Furthermore, the authors employed a T2 contour, and finally, this investigation was based on synthetic IRN sounds which did not exhibit distinct formant relationship as found for vowels or French horn sounds. Thus, further studies with extended sets of stimuli including natural as well as IRN sounds are needed to further assess these relationships.

### Location of sustained field generators

In the current study, we found a group specific difference regarding the location of the SF generators: the average position of the SF dipoles in the Chinese group was found to be closer to the vowel- and pitch-specific region [35], whereas for the Germans listeners, the position was slightly shifted to the posterior border of HG [40]. Comparison of our results with a recent 4-dipole model shows [58], however, that the SF of the German speakers in the current investigation is localized rather closely to pitch related positions. The discussion concerning source location differences should also take into account that dipole fitting was based on a spherical head model. Although such models allow to infer MEG dipole locations in the temporal lobe with sufficient accuracy [39], it is important to keep in mind that head and brain shapes of people of Chinese and of Central European descent differ substantially in length and width [59–61]. Thus, the shift to more posterior sites might be an effect of a biased sphere fitting procedure. However, a possible bias due to such a shift might not be expected to interfere with the findings of the current investigation, since the model free RMS values mirror closely the results of the dipole analyses. Nevertheless, future investigations should rely on realistic head models in order to take into account individual cortex geometry.

### Lateralization

In our experiment, where subjects listened passively with distracted attention to stimuli, the SF did not show a general lateralization effect, neither for *f*_0_ contours of the Mandarin tones in experiment 1, nor for the flat *f*_0_ pattern of the French horn sound. This result seems to contradict the findings of Eulitz et al. [24], who observed larger vowel-evoked sustained responses over the left hemisphere. However, the difference between the studies might be due to the specific task in Eulitz’ experiment where subjects had to solve a duration discrimination task. Such attentional effects were shown to enhance sustained responses, especially in the left hemisphere [62, 63].

The missing group by lateralization interaction effect seems to stand in contrast to recent EEG data where a right hemisphere lateralization for the transient pitch related onset responses evoked by Mandarin tone contours was observed in Chinese listeners [31]. Based on earlier finding regarding the specific contribution of the left hemisphere to pitch processing [64], these effects were associated with specific pitch mechanisms influenced by language experience. However, it is important to note that, in the same investigation, the subsequent contour evoked response referred to as the Nb component did not result in a comparable rightward lateralization in Chinese listeners. Furthermore, it is not clear how to relate these transient components to the sustained responses of the current study that were assessed using different stimulation procedures. First, overall onset responses represent complex mixtures of energy und pitch related generators [14, 58]; second, as shown in Figs 2–4, the major effects in our investigation were elicited after 300 ms and lasted several hundred milliseconds. Although N1m and SF are often found to be generated in similar cortical regions [15], the overall N1-SF pattern of experiment 1 supports the assumption that the SF is more strongly coupled to language related effects; thus, the specific generators in AC are probably driven by the strong regularity of sounds, as found in earlier SF recordings [26, 35]. Moreover, a recent AEP study on categorial speech perception employed source analysis to investigate pitch specific responses [54]. While, in this study, the source waveforms evoked by T2/T3-contours resulted in comparable group effects as outlined above, a lateralization effect was not observed.

### Dissociation between SF and N400

Due to the rather short stimulus duration in our experiments, the SF morphology did not exhibit some continuous DC offset as usually observed for regular sounds with long duration [35]. Instead, we observed a prominent broad peak at about 400 ms, followed by a decay up to 1000 ms. This closely resembles the SF waveform morphology observed by Eulitz et al. [24] who used synthetic vowels of a comparable length to investigate the different activations evoked by a sine tone and vowels. It is important to note that this broad deflection with a peak at about 400 ms does not represent the N400 component which has been observed in experiments on semantic processing [65]. This conclusion is based on several arguments: The signal difference between the two language groups depicted in blue in Fig 4g does not exhibit a steep valley 400 ms after onset, but a broad plateau ranging from 300 to 550 ms including a very slow decay. Moreover, the waveform amplitude shows an integrative behavior such that the integrated SF was linearly correlated with stimulus duration, as shown in Fig 3c. The SF observed in the current investigation can be well described by an equivalent dipole located in the lateral aspect of HG, and the position of the SF generators has been shown to be stable, irrespective of the fit interval that was chosen to model the data (either 300–600 ms or 500–800 ms after stimulus onset, as shown in Fig 3e). Furthermore, we did not observe any lateralization effect for vowels and syllables in both groups. Finally, since our items were presented in random sequences and subjects were listening passively with distracted attention from the auditory input, a N400 signal might not be expected to emerge (cf. the typical features of the N400 signal as described by several authors [66–72]).

### Transient activity

In contrast to the sustained response, we observed no significant global group-specific difference in magnitude for the transient N100m, see Table 1, line 5a. However, a highly significant group-specific difference was observed for the correlation coefficients. There was a strong correlation between the average N100m-signal strengths of the two groups evoked by stimuli that were meaningless for both groups, but no significant correlation was observed for the seven syllables that are meaningful to Chinese listeners. This difference can be traced back to the following feature: N100m responses to signals, which are meaningful for the Chinese, were significantly larger for this group than for Germans, whereas there was no difference in the N100m response strengths to those stimuli, which were meaningless for both groups. This can be explained in the following way: For meaningless syllables, only physical properties determine the response, and therefore, there is a strong correlation of the responses to these signals between groups. For Germans all stimuli were meaningless, but the vowels were acoustically more salient than the syllables, since the latter start with a consonant. Therefore, the N100m signal evoked in Germans was stronger in response to vowels than for the syllables. The fact that in the Chinese group, the vowels and syllables lead to comparable responses shows that another factor apart from the physical properties plays a substantial role in the generation of the N100m in these listeners. Since this additional factor is related to semantic aspects, it might be related to the very fast (bilateral) semantic filter outlined above. Recently, MacGregor et al. [46] provided evidence for the existence of such an ultra-rapid lexical process.

## Conclusions

Although, as pointed out by Manca and Grimaldi [73], neither the N1 nor the SF can be used to derive a speech-specific representation, the current experiments show that the SF might be employed to track AC-STG interactions in lateral HG. In order to reveal more generalized findings, future investigations should apply “roving” designs, i.e., investigate language related sustained responses evoked by much more different speakers as carried out by the pioneering work of Mesgarani et al. [53]. Such material should be contrasted with nonnative *f*_0_ contours to bridge the gap between investigations which employed either natural or synthetic stimuli. Taken together, the current data provide evidence that the SF is involved in a complex feedforward-feedback loop and reflects language specific properties at higher levels in the temporal lobe. This neurophysiological finding is of special importance since the fMRI BOLD response seems to be dominated by more transient activity [74].
